# Iatrogenic Infective Endocarditis in Hemodialysis Patients: A Case Report and Review of the Literature

**DOI:** 10.1155/2022/8500299

**Published:** 2022-06-29

**Authors:** Ali Oullaï, Edouard Cubilier, Mohamed Tayeb Salaouatchi, Maxime Taghavi, Yasmin Zouggari, Joëlle Nortier, Maria Do Carmo Filomena Mesquita

**Affiliations:** Department of Nephrology, Brugmann University Hospital, Université Libre de Bruxelles, Brussels, Belgium

## Abstract

Foreign bodies such as implanted cardiac devices are susceptible to infections and may be involved in infective endocarditis. Exposure to pathogens, by frequent use of intravascular accesses for hemodialysis (i.e., catheters or fistulas), combined with high rates of degenerative heart valve diseases in hemodialysis patients, both favor the development of infective endocarditis in this population. The mitral and aortic valves are predominantly implicated in endocardial infections. The involvement of both mitral and tricuspid valves is rare in the general population but can occur in hemodialysis patients with implanted cardiac devices. Infective endocarditis is associated with high morbidity and mortality rates among hemodialysis patients, mostly because of the complications of septic emboli. Prevention, prophylaxis, and early diagnosis of endocarditis can be lifesaving in this fragile population. We report a case of right and left heart methicillin-sensitive *Staphylococcus aureus* endocarditis with cerebral septic emboli in an elderly hemodialysis patient carrier of an arteriovenous fistula and an ipsilateral nonleadless pacemaker.

## 1. Introduction

Cardiovascular diseases and infections are the two main causes of mortality in hemodialysis (HD) patients [1]. The prevalence of infective endocarditis (IE) in chronic HD patients is 2.9% [2], and its incidence is 50 to 60 times higher than in the general population [3].

HD patients are at higher risk of IE due to their intrinsic fragility related to older age and multiple comorbidities, such as cardiovascular diseases and hyperuricemia-induced immunosuppression [4], in addition to a high exposure to pathogenic microorganisms during HD sessions due to repeated manipulations of their vascular access [1]. Moreover, mortality rates of IE in the HD population reach 25 to 77%, which is higher than the general population [1]. Sadeghi et al. reported in-hospital IE death rates and long-term death rates as 45.6% and 29.5% of cases, respectively [2].

Other risk factors for IE in the general population include the presence of implanted cardiac devices as well as the absence of antibiotic prophylaxis during dental and surgical procedures [5]. Methicillin-sensitive *Staphylococcus aureus* (MSSA) is the main pathogen involved in IE as reported by several previous studies [6]. IE is often associated with several complications such as systemic embolism, among which brain septic emboli are the most common, with an incidence of 65% in MSSA-related IE. Such complications were associated with longer ICU stays and higher mortality rates [7].

## 2. Case Presentation

**Figure 1:**
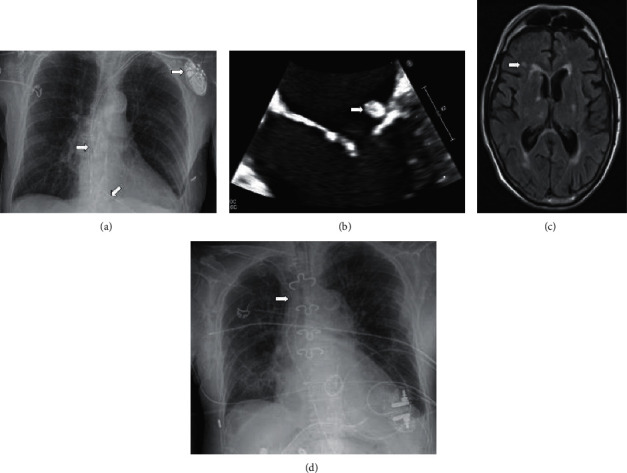
(a) Chest radiography with implanted pacemaker, (b) transesophagal echography, (c) MRI cerebral showed the emboli, and (d) chest radiography with external pacemaker.

We report a case of a 77-year-old hemodialyzed man with a medical history of congenital anomalies of the kidney and urinary tract (CAKUT)-related ESRD requiring HD. He underwent a left brachial radio-radial AVF and began dialysis sessions in our center 6 years ago. The patient was also known for a severe chronic obstructive pulmonary disease (COPD) (Gold IV) due to smoking and chronic alcoholism. Moreover, he benefitted, two years ago, from a dual chamber St Jude Assarity Magnetic Resonance Imaging (MRI) PM placement for an auriculoventricular block of 2 : 1, with leads placed in the right heart via the left jugular vein in the right atrium and right ventricle, respectively (Figure 1(a)).

The patient was admitted to the emergency room for fever. A clinical examination revealed a debilitated patient with a fever (40°C) and chills. Examination of the AVF showed no sign of infection, and an oral examination did not show decayed teeth. No invasive dental, respiratory, gastrointestinal, or genitourinary procedures were performed for the last year.

Relevant laboratory findings displayed an elevated inflammatory marker C-Reactive Protein of 408 mg/L (reference values (RF): <0.5 mg/dL), a leukocytosis of 11 260/*µ*L (RF: 3.50–11.00 [×$\hat{103}$/*µ*L]) with 10.360/*µ*L neutrophils (RF: 1.50–6.70 [×$\hat{103}$/*µ*L]), ion disorders hyperkaliemia at 5.6 mmol/L (RF: 3.4–4.5 mmol/L), hypercalcemia at 2.56 mmol/L (RF: 2.20–2.55 mmol/L), and hyperphosphatemia at 2.27 mmol/L (RF: 0.75–1.39 mmol/L). Four sets of blood cultures were collected, all of which were positive for Gram-positive cocci identified as MSSA.

The thoracic-abdominal computed tomography (CT) scan performed did not show any acute abnormalities, despite COPD lung lesions, polycystic kidneys, and noninflammatory diverticulosis.

Initial antibiotic treatment consisted in intravenous (IV) ceftazidime and vancomycin, prior to blood culture results, and was then switched to IV flucloxacillin sodium according to the antibiotic sensitivity spectrum, with an adjusted dose for HD patients (2 g four times a day). Antibiotic doses were then increased to six times a day, because of increased laboratory inflammatory markers and persisting bacteremia five days after admission.

The trans-esophageal echocardiography (TEE) showed vegetations on the tricuspid valve (18 × 10 mm), on the mitral valve's posterior leaflet (8 mm), and on the atrium lead of the PM (<10 mm) (Figure 1(b)).

Four days after admission, the patient presented with delirium and left upper limb paresis. A cerebral MRI was performed and showed multiple ischemic lesions with embolic features (Figure 1(c)).

The pacemaker box and its leads were removed on the 10^th^ day and a temporary external pacemaker was placed (Figure 1(d)). There were no signs of infection on direct examination. The whole device was put into culture and the results were negative. The patient was then transferred to the cardiology unit for cardiac monitoring.

Despite two weeks of a well-conducted antibiotic therapy, the control of TEE showed an increase in the size of the mitral vegetation (15 mm), in addition to the appearance of an abscess of the aortic annulus, which required cardiac surgery.

On the 17^th^ day after admission, the patient underwent a mitral valve replacement by bioprosthesis and a tricuspid valvuloplasty. The surgical report described a residual vegetation on the posterior valve of the tricuspid valve where the ventricular lead used to be in place and a massive invasion of the mitral valve by a voluminous vegetation. The patient was then transferred to the intensive care unit (ICU) for postoperative monitoring. During the ICU stay, the patient presented with hemodynamic instability due to a global cardiac dysfunction which culminated in an acute pulmonary edema, and a sepsis related to a *Pseudomonas aeruginosa* ventilator-associated pneumonia (VAP). HD was continued thrice a week and supplementary HD was administered whenever necessary.

**Figure 2:**
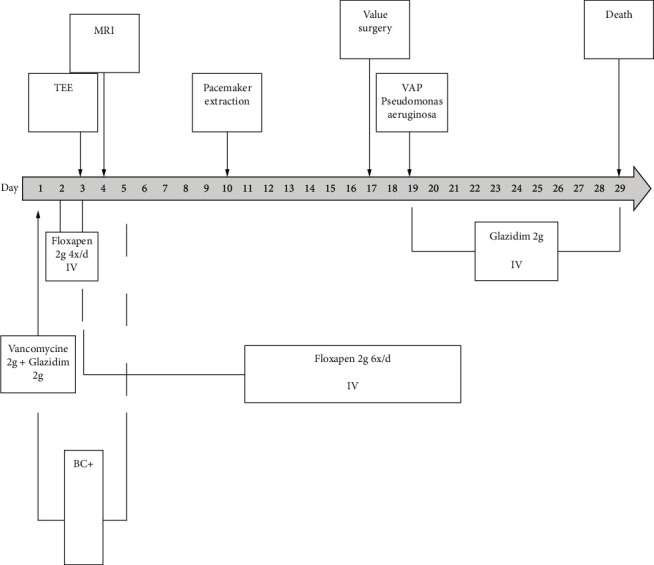
Time line of clinical course, diagnostics, interventions, and outcomes. BC: blood culture, TEE: transoesophageal echography, MRI: magnetic resonance imaging, and VAP: ventilator-associated pneumonia.

After clinical stabilization, the patient was transferred back to the cardiology ward with a temporary pacemaker in await for the definitive leadless PM which had been ordered. Unfortunately, the patient died on the 29^th^ day of hospitalization because of recurrent cardiogenic shocks. The timeline of clinical course, diagnostics, interventions and outcome are shown in Figure 2.

## 3. Discussion

The risk factors for endocarditis in HD patients are well known. Nevertheless, prevention and rapid diagnosis are essential for optimal management in these fragile patients.

Chronic HD patients are vulnerable to IE because of frequent valvular degeneration secondary to the cardiovascular complications of ESRD [1]. These complications imply fluid overload, uremic cardiomyopathy, secondary hyperparathyroidism, anemia, altered lipid metabolism, myocardial hemodynamic stress due to HD, and accumulation of gut microbiota uremic toxins like trimethylamine N-oxidase [8]. HD patients often present with arterial hypertension, which raises mechanical stress on the valves of the left heart by increasing cardiac postcharge, thus accelerating valve deterioration [1]. Moreover, valve damage can result from “jet lesions” due to turbulent blood flow during HD or by repeated IV drugs that are commonly given during dialysis sessions [9]. Indeed, our patient, who underwent HD for 6 years, had known calcifications on both the aortic and mitral valves.

In HD patients, the mitral and aortic valves are predominantly involved in IE, with a respective prevalence of 80 and 100% of IE cases [1]. The left heart is a high-pressure system that tends to deteriorate the mitral and aortic valves faster, which therefore promotes valve calcification in the context of frequent calcium-phosphate disorders, as seen in ESRD patients. Right-sided IE is rare and its incidence varies from 0% to 26% depending on different studies, with a predominant implication of the tricuspid valve [1]. In our patient, we observed simultaneous right (tricuspid) and left (mitral) involvement. As the leads of the PM were in the right heart and were infected, the formation of vegetation on the tricuspid valve could have followed. The left heart involvement may have occurred as a result of an embolus via the pulmonary circulation.

The principal source of infections in HD patients is their vascular access, which can be either a catheter or an AVF [10]. AVF induces fewer infectious complications compared to other accesses. AVF-related infection occurs in 2–4% of this population, with an incidence of 0.018/100 per access day [11]. Clinical examination of the AVF by the patient, nursing staff, and medical staff should be routinely done. In our patient, on admission and thereafter, meticulous examination of the AVF showed no sign of infection. Nonetheless, its repeated punctures during HD sessions could have been the portal of entry.

Cardiac implantable devices such as PM, implantable cardiovascular defibrillators, or cardiac resynchronization therapy devices are vulnerable to infections [12]. PM-related infections have been estimated to be 0.77% for their initial implantation and 2.08% for revision or replacement procedures [13]. Device infection can occur because of skin infections which may colonize the pocket of the device or by hematogenous implantation of bacteria [13]. In our patient, the insertion site of the PM bared no sign of infection and there was no report of any infectious complication related to the device since its installation two years ago.

In HD patients, cardiac devices should be placed on the contralateral side of the vascular access due to the increased risk of venous stenosis and infection of the device [14]. Unfortunately, our patient's PM was placed on the ipsilateral side of the AVF because puncture of the contralateral jugular vein was not technically possible. He had a left radio-radial AVF, and the leads of PM were introduced through the left cephalic vein in the right side of the heart. This setup increases the risk of hematogenous bacterial colonization on the device because of the direct vascular contact of the microbes with the PM. Indeed, we hypothesized that repeated punctures of the AVF could have silently brought bacteria to the PM.

In our patient, PM was removed a week before surgery. We observed an increase in the size of the mitral valve vegetation despite two weeks of adequate antibiotic therapy. The vegetations seen on the valves were much bigger than those seen on the leads of the PM. The bacteria were probably more adherent to the endothelium of the sick valve than to the biofilm of the lead. This could explain the bigger lesions on the mitral valve compared to the flat surface of the lead of the PM [15].

Blood cultures obtained before the beginning of any antibiotic treatment are an important part of the diagnostic procedure. They must be repeated daily until they come back negative to determine the first day of the 6 weeks of antibiotic therapy [16]. In addition to antibiotics, device removal is indicated if it is infected, or in the case of IE, or a Gram-positive bacteremia [17]. Leadless PM reduces the risk of device infection and central venous stenosis because no invasive intravascular intervention is needed to install the leads, and leadless PM infections are very rare [13]. This could be due to the absence of a subcutaneous pocket and transvenous leads, the small surface area of this device, and the parylene coating of the leadless PM [13]. In fact, parylene reduces bacterial adherence and has antibacterial properties compared to either polyurethane or bare titanium which composes the pulse generators of traditional PM [13]. Our patient did not benefit from this novel device at that time because leadless PM was not available two years ago in our center.

IE can have local and distant complications. Heart failure in IE can occur because of a valvular dysfunction directly caused by the vegetation [18] or a peri-annular extension of the infection which then urges the need for surgical procedures and raises mortality rates [18]. Distant IE complications on a remote site occur via septic emboli. The destination of septic emboli depends on the heart valve involved; right-side IE frequently causes pulmonary septic emboli, whereas left-side IE causes systemic septic emboli [7]. In a multinational study, 44.4% of patients with IE had major embolic events, predominantly in the brain (26.3%), as it occurred in our patient [18].

General septic emboli are best diagnosed by fluorodeoxyglucose positron emission tomography (FDG PET-CT), but this exam cannot detect cerebral emboli because of the intense cerebral capture of the FDG [19]. MRI is therefore the gold standard for the diagnosis of cerebral septic emboli because of its high sensitivity compared to CT-scan [20].

Treatment of IE in an HD population should combine preventive strategies such as reducing hospital-acquired bacteremia, good oral hygiene, antibiotic prophylaxis according to the local guidelines when required, especially for dental and other surgical procedures, and the use of antibacterial coating materials in implantable devices when available. Special attention is required in patients with implanted cardiac devices that could be silently infected. In order to improve diagnosis, a high index of systematic clinical suspicion of device infection is required. These patients should therefore be educated on related signs and symptoms, an adequate microbiological assessment must be made rapidly in order to start empiric treatment, and a TEE must be scheduled hastily. Optimal management of these fragile patients with the help of an IE-devoted team is advisable. Tailored antibiotic therapy and early surgery when required, as well as monitoring for complications, are necessary in order to face the challenges of the dreaded IE complications [5].

Unfortunately, our patient died on the 29^th^ day of hospitalization and 12 days after cardiac surgery. The in-hospital mortality rate of HD patients with IE is up to 45.6% [2]. The prognosis of patients with IE is determined by four main factors: patient characteristics, the occurrence of IE-related cardiac and noncardiac complications, the infecting organism, and the echocardiographic findings. In patients with three or more of these factors, as in the case of our patient, the in-hospital mortality rate reaches 79% [21].

In addition, our patient had signs of persistent infection as he presented with a fever and persistent positive blood cultures for five days after starting the antibiotics. The persistence of positive blood cultures after 48–72 h of antibiotic therapy has been recently associated with a higher risk of hospital mortality [22], which suggests, in fact, that early surgery should have been considered in our patient.

Early surgery was indeed considered in our patient, but the occurrence of symptomatic septic emboli and the clinical stability of the patient motivated our cardiac team to delay the surgery. In patients with a good response to antibiotics, surgery can be postponed for 2–4 weeks under close monitoring of the aortic abscess by serial TEE [23].

Finally, the control of TEE after two weeks of antibiotics in our patient showed an increase in the size of the mitral vegetation, in addition to the appearance of an abscess of the aortic annulus, which indicates locally uncontrolled infection and then urged the need for surgical procedure. However, it is known that the results of surgery when the reason for the procedure is uncontrolled infection are worse than when surgery is performed for other reasons [21].

## 4. Conclusion

Awareness about IE is crucial in clinical practice given its high morbidity and mortality in HD patients. In this population, the risk of IE increases because of high rates of valvular damage as a consequence of metabolic and cardiovascular complications of ESRD. Vascular access is often the source of IE even in the absence of clinical signs of infection.

Implanted cardiac devices are risk factors for endocarditis and should be placed on the contralateral side of HD patients' vascular access whenever possible. Leadless PM should be considered in HD patients, but the cost, the availability, and the expertise can limit its prescription.

Regular planning of antibiotic prophylaxis during dental and different surgical procedures should be in the good practice guidelines of all the dialysis centers.

For optimal management of IE, a devoted IE team of nurses and clinicians is advised in order to prevent and treat IE.

Endocarditis in hemodialysis: key points
Clinical suspicion and early diagnosis are necessary
Careful monitoring of the vascular access sites is important
Antibiotic prophylaxis in the case of dental and all surgical procedures is recommended
If an implantable cardiac device is required:
    Implant on the contralateral side of the device when possible
    Use a leadless pacemaker if available
    Remove the device if MSSA bacteremia, valvular, or device infection occurs
Look for septic emboli and discuss appropriate treatment
Work with a dedicated IE team
